# Sex‐specific mitochondrial dynamics and mitophagy response to muscle damage

**DOI:** 10.14814/phy2.15230

**Published:** 2022-05-25

**Authors:** Hui‐Ying Luk, Nigel C. Jiwan, Casey R. Appell, Danielle E. Levitt, Jakob L. Vingren

**Affiliations:** ^1^ Texas Tech University Lubbock Texas USA; ^2^ Louisiana State University Health Sciences Center New Orleans Louisina USA; ^3^ University of North Texas Denton Texas USA

**Keywords:** fission, fusion, OPA1, Parkin, resistance exercise, testosterone

## Abstract

Muscle damage imposes stress on mitochondria resulting in mitochondrial fusion, fission, and mitophagy. Testosterone is a regulator of these processes. However, no study has examined the effect of sex‐specific resistance exercise (RE)‐induced hormonal response on mitochondrial dynamics and mitophagy after muscle damage in untrained men and women. Untrained men and women performed two sessions of 80 unilateral maximal eccentric knee extensions (ECC) followed by upper‐body RE (ECC+RE) aimed to induce hormonal changes and maintain a similar lower body demands between conditions or 20 min seated rest (ECC+REST). Vastus lateralis samples were analyzed for gene and protein expression of OPA1, MFN1, DRP1, PINK1, and Parkin at baseline (BL), 12 and 24 h. Testosterone area under the curve was greater for ECC+RE than ECC+REST in men and was greater in men than women for both conditions. A significant time × sex × condition effect was found for Parkin protein expression. At 12 and 24 h, Parkin was lower for ECC + REST than ECC + RE for men; whereas, Parkin was increased at 24 h for women regardless of condition. A significant time effect was found for OPA1 protein expression increasing at 12 and 24 h. A significant time × sex × condition effects were found for *MFN1*, *DRP1*, and *PINK1* gene expression with increases at 12 h in men for ECC + RE. A significant time × sex effect was found for *OPA1* gene expression with a decrease at 12 h in men, and 12 h expression in men was lower than women. RE‐induced hormonal changes promoted expression of fission, fusion, and mitophagy markers in men. With muscle damage, regardless of condition, expression of inner mitochondrial membrane fusion markers are promoted in both sexes; whereas, those for mitophagy were promoted in women but reduced in men.

## 1 INTRODUCTION

Skeletal muscle damage and regeneration is an inevitable part of exercise adaptation. In exercise‐induced muscle damage, both mechanical and chemical damage impose stress on different organelles within the skeletal muscle, including the mitochondria (Ying et al., 2021). With muscle damage‐associated mitochondrial damage, the processes of mitochondrial fission, inner and outer mitochondrial membrane (IMM and OMM) fusion, and mitophagy are altered to reorganize the network or remove damaged mitochondria (Youle & Bliek, 2012). Dysregulated mitochondrial dynamics (fusion and fission) and mitophagy can result in the accumulation of damaged mitochondria and oxidative stress, thus leading to impaired skeletal muscle health (Sebastián et al., 2017).

The mitochondrial network is constantly reorganizing through IMM (optic atrophy gene 1: OPA1) and OMM (mitofusin: MFN) fusion and fission (dynamin‐related protein 1: DRP1) to maintain healthy mitochondria and promote efficient energy production (Hoitzing, et al., 2015; Yu & Pekkurnaz, 2018). Following fission, damaged mitochondria are removed via mitophagy (PINK1 and Parkin) (Narendra, et al., 2010; Youle & Bliek, 2012). These processes are essential in maintaining muscle mass (Dulac et al., 2020; Tezze et al., 2017), myofibril contractility (Gouspillou et al., 2018), muscle force production (Gouspillou et al., 2018; Tezze et al., 2017), and preventing mitochondrial dysfunction (Chen et al., 2010; Gouspillou et al., 2018) in vivo and in vitro. In response to 100‐min downhill running (resulting in substantial exercise‐induced muscle damage), mitochondrial vacuolar degeneration and the absence of cristae were observed in mouse skeletal muscle from immediately to 24 h after the run, and the severity of the altered mitochondrial phenotype was alleviated after 48 h (Ying et al., 2021). These findings illustrate that eccentric exercise‐induced damage includes damage to the structure of the mitochondria; however, the understanding of muscle damage on markers of mitochondrial network remodeling and mitophagy is limited. Besides eccentric exercises, an unaccustomed bout of RE can also induce muscle damage leading to damaged mitochondria (Kitaoka et al., 2015; Mesquita et al., 2020). In response to an unaccustomed bout of RE, IMM fusion (OPA1) increased with no changes in fission and mitophagy proteins were observed at 24 h in older adults (Mesquita et al., 2020). *On the contrary*, *an increase in fission (DRP1^Ser616^) with no changes in fusion proteins was observed at 24 h in young male rats* (Kitaoka et al., 2015). *Despite the inconsistent results observed in the mitochondrial response to RE*, *these studies demonstrated that mitochondrial remodeling occurs in damaged muscles*.

The signal to initiate mitochondrial network remodeling could primarily be regulated by the increased demand for efficient energy production (Liesa & Shirihai, 2013); however, similar to other cellular responses, hormones can regulate nuclear genes that encode mitochondrial transcription factors. For example, C2C12 muscle cells incubated with testosterone had increased nuclear respiratory factor 1 (*NRF1*) and *OPA1* gene expression (Pronsato et al., 2020). Besides the role of NRF‐1 in mitochondrial biogenesis and fusion (Pronsato et al., 2020), Lu et al. reported an NRF‐1 binding site in the promoter regions of mitophagy genes (*PINK1* and *Parkin*) (Lu et al., 2020), where the overexpression and knockdown of *NRF1* resulted in upregulation and downregulation of mitophagy genes (i.e., *PINK1* and *Parkin*), respectively (Lu et al., 2020). Furthermore, Guo et al. compared mitochondrial dynamics and mitophagy gene and protein expression after low‐intensity endurance training in very old male mice supplemented with or without testosterone (Guo et al., 2012). In response to exercise training, the results showed greater gene expression of *PINK1*, *OPA1*, and *DRP1* and greater protein expression of DRP1 and MFN2 in the mice with testosterone than mice without testosterone supplementation (Guo et al., 2012). Although this study did not include a group with testosterone supplementation alone (i.e., no exercise), this result demonstrated the potential importance of testosterone and exercise on mitochondrial dynamics and mitophagy. High volume RE is a potent stimulator of testosterone (Vingren et al., 2010) *and the magnitude of RE*‐*induced testosterone increase was greater in men than women* (Weiss et al., 1983). Given that basal testosterone concentration and RE‐induced increases in testosterone concentrations are greater in men than women, it is possible that this sex‐specific hormonal response could affect the mitochondrial response to muscle damage.

**FIGURE 1:**
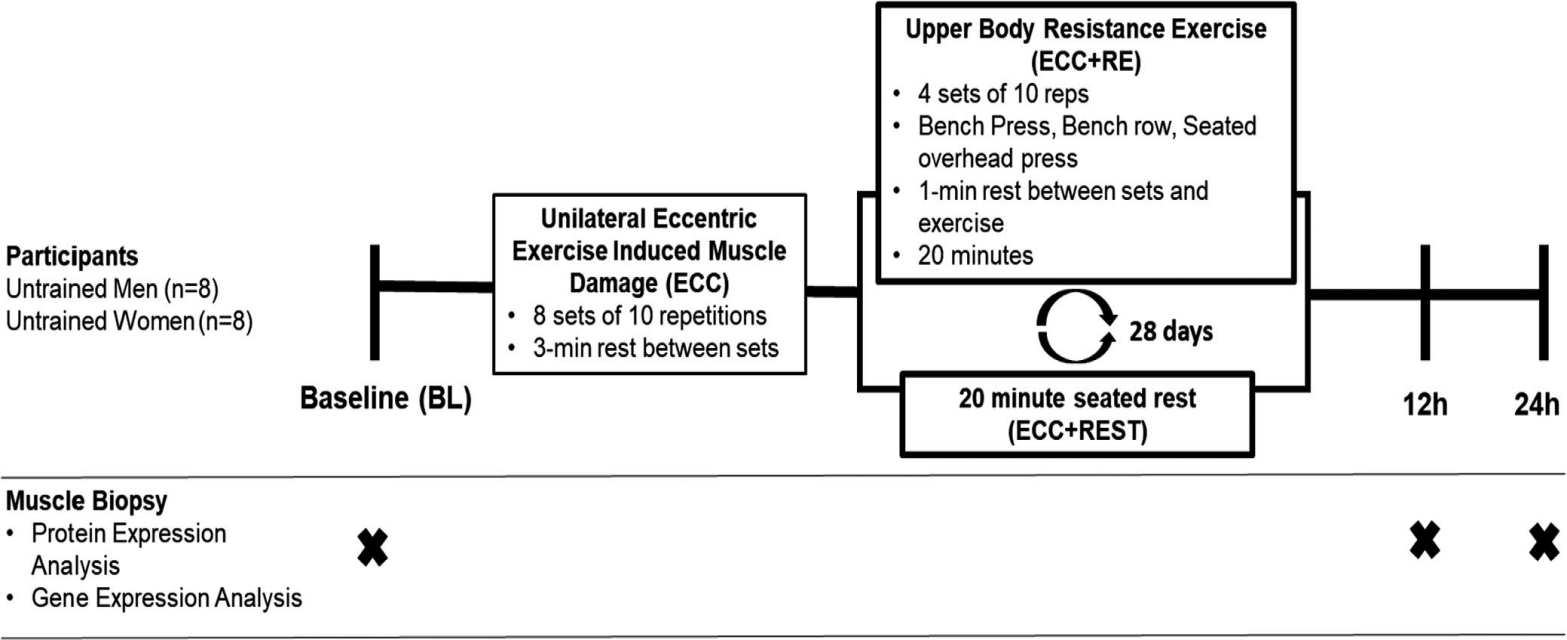
Study design

Healthy, functional mitochondria could serve an important role in muscle regeneration after muscle damage (Sin et al., 2016), and previous reports demonstrate that hormonal stimuli influence mitochondrial dynamics and mitophagy (Guo et al., 2012; Hu et al., 2021; Lu et al., 2020; Pronsato et al., 2020). However, no prior research has investigated the effect of the sex‐specific RE‐induced hormonal changes (e.g., testosterone) on the mitochondrial response after muscle damage. Therefore, the purpose of the study was to examine the impact of the RE‐induced hormonal response on markers of mitochondrial dynamics and mitophagy after muscle damage in young untrained men and women. To achieve this purpose, a randomized cross‐over design was utilized (Figure 1). *Participants completed maximal unilateral knee eccentric contraction exercise (ECC*; *aimed to induce muscle damage) immediately followed by either upper*‐*body RE (ECC *+* RE*; *aimed to elicit systemic hormonal changes and maintain a similar lower body demands between conditions) or seated rest (ECC* +* REST)*. *Blood samples were collected to confirm that the upper*‐*body RE elicited a hormonal response*, *while muscle samples were collected to examine intramuscular gene and protein expression of selected markers of fission (DRP1)*, *IMM fusion (OPA1)*, *OMM fusion (MFN1)*, *and mitophagy (PINK1 and Parkin)*. *With the importance of testosterone on mitochondrial network remodeling*, *this design will provide the means to determine the role of RE*‐*induced testosterone on mitochondrial response to muscle damage between sexes*.

## 2 RESULTS

### 2.1 Effect of RE‐induced hormonal changes on markers of mitochondrial dynamics after muscle damage in men and women

To examine the effects of RE‐induced hormonal changes on markers of mitochondrial dynamics and mitophagy after muscle damage, we analyzed muscle samples from a subset of participants (men: *n* = 8; women: *n* = 8), and the details of the study design (Luk et al., 2019) and participants’ descriptive data (Luk et al., 2021) have been previously reported. The area under the curve (AUC) for testosterone, growth hormone, and cortisol concentrations was calculated. These hormonal data were published previously (Luk et al., 2021). Briefly, for men, testosterone and growth hormone AUC was greater for ECC + RE than ECC + REST. Additionally, testosterone AUC was greater in men than women for both conditions, while growth hormone AUC for ECC + RE was greater in men than women for ECC + RE and ECC + REST. For women, growth hormone AUC was greater for ECC + RE than ECC + REST. Lastly, for both men and women, cortisol AUC was greater for ECC + RE than ECC + REST.

To assess skeletal muscle mitochondrial dynamics markers, we collected muscle samples at BL, 12 (12 h), and 24 (24 h) after ECC. Intramuscular gene and protein expression of selected markers were used to examine fission (DRP1), IMM fusion (OPA1), and OMM fusion (*MFN1*).

**FIGURE 2:**
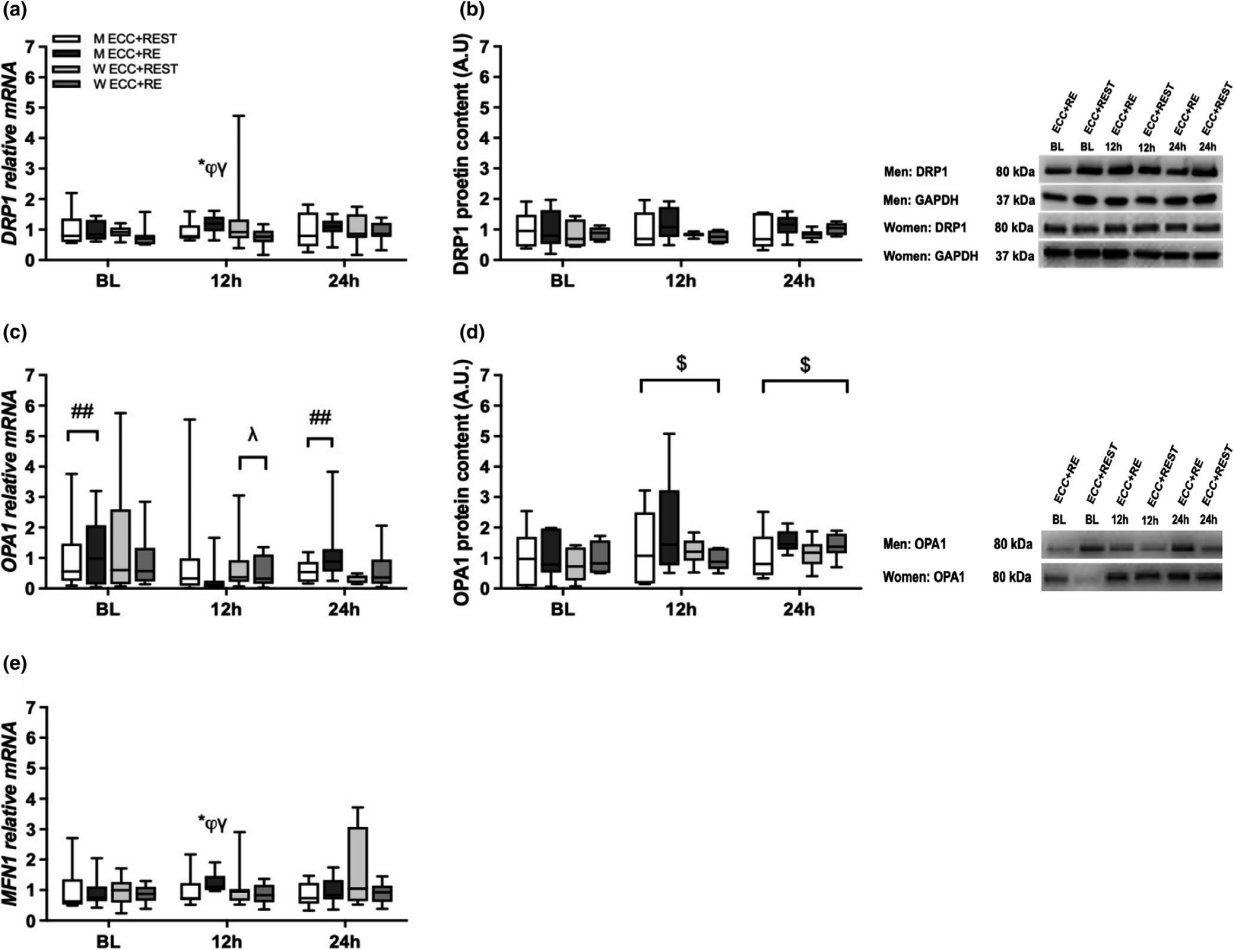
Gene and protein expression analyses for (a and b) DRP1, (c and d) OPA1, and (e) MFN1. For gene expression analysis [men (M): *n* = 8; women (W): *n* = 8], data were normalized to Men ECC + REST at BL. A time × sex × condition interaction effect was observed for *MFN1* and *DRP1*. A time × sex interaction effect was observed for *OPA1*. For protein expression analysis [M: *n* = 6; W: *n* = 6], data were normalized to men ECC + REST at BL. A main effect of time was observed for OPA1. The box and whisker plots display an example of BL, 12 h, and 24 h loading gene and protein expressions for DRP1 and OPA1. The corresponding GAPDH for an individual subject between conditions (ECC + RE and ECC + REST) were run on the same gel for men and women is displayed in Figure 2b. MW, molecular weight (kDa). *p < 0.05 versus ECC + REST. φ*p* < 0.05 versus BL and 24h. γ P< 0.05 versus Women ECC+RE. ##*p* < 0.05 versus 12 h (collapsing for condition). φ *p* < 0.05 versus Men (collapsing for condition). $*p* < 0.05 versus BL (collapsing for condition and sex)

Significant (*p* ≤ 0.05) time × sex × condition interaction effects were observed for *MFN1* and *DRP1* gene expression. For men in ECC + RE, *MFN1* (Figure 2e) and *DRP1* (Figure 2a) gene expression increased from BL to 12 and decreased from 12 to 24 h, which resulted in a greater expression than for women in ECC + RE and men in ECC + REST. In addition, a significant time × sex interaction effect was observed for *OPA1* gene expression. Regardless of condition, *OPA1* decreased from BL to 12 h and then increased to 24 h for men, which resulted in a lower expression at 12 h than for women (Figure 2c).

For protein expression, there were no significant changes observed for DRP1 (Figure 2b). However, for protein expression, a significant main time effect was observed for OPA1, increasing from BL to 12 and 24 h (Figure 2d).

### 2.2 Effect of RE‐induced hormonal changes on markers of mitophagy after muscle damage in men and women

**FIGURE 3:**
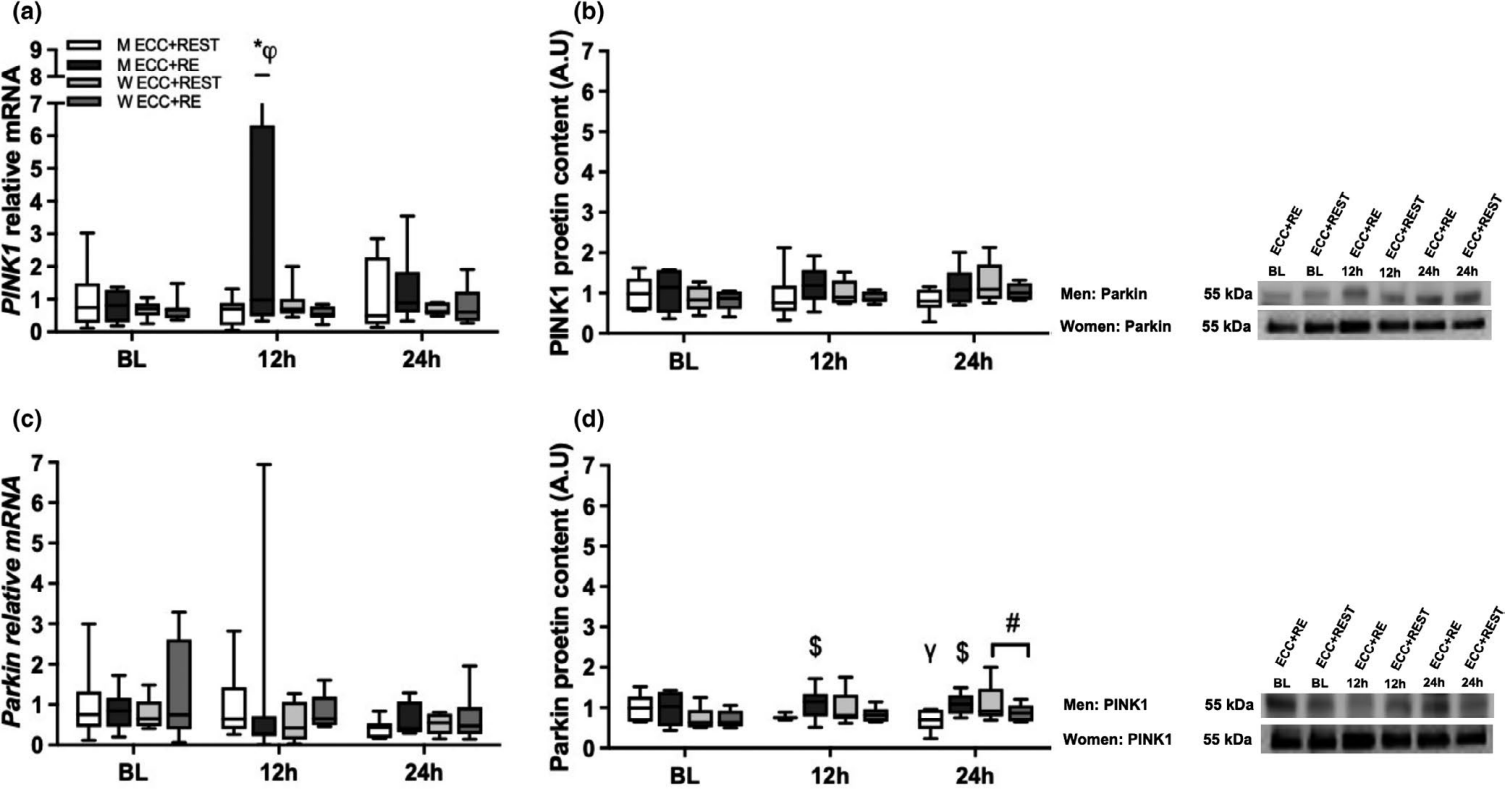
Gene and protein expression analysis for (a and b) PINK1 and (c and d) Parkin. For gene expression analysis [men (M): *n* = 8; women (W): *n* = 8], data were normalized to Men ECC + REST at BL. A time × sex × condition interaction was observed for *PINK1*. For protein expression analysis [M: *n* = 6; W: *n* = 6], data were normalized to Men ECC + REST at BL. A sex × condition × time interaction was observed for Parkin. A time × sex interaction was observed for Parkin. A condition × sex interaction was observed for Parkin. The box and whisker plots display an example of BL, 12 h, and 24 h loading gene and protein expressions for PINK1 and Parkin (see Figure 2b for the corresponding GAPDH). MW, molecular weight (kDa). * *p* < 0.05 versus ECC + REST. φ *P* < 0.05 versus BL. $ *p* < 0.05 versus ECC+REST. γ P< 0.05 versus BL. # *p* < 0.05 versus BL

To assess skeletal muscle mitophagy, intramuscular gene and protein expression of selected markers of mitophagy (PINK1 and Parkin) were examined. A significant (*p* ≤ 0.05) time × sex × condition interaction effect was observed for *PINK1* gene expression. In men, *PINK1* gene expression was increased from BL to 12 h for ECC + RE and was greater for ECC + RE than for ECC + REST at 12 h (Figure 3a). Results indicated no significant (*p* ≥ 0.05) differences for *Parkin* gene expression (Figure 3c).

For protein expression, significant (*p* ≤ 0.05) time × sex × condition and time × sex interaction effects were observed for Parkin. In men, Parkin protein expression decreased from BL to 24 h for ECC + REST (Figure 3d), which resulted in a lower expression than ECC + RE at 12 and 24 h. Additionally, in women, Parkin protein expression increased from BL to 24 h (Figure 3d). No significant differences were observed for PINK1 protein expression (Figure 3b).

### 2.3 Hormones association with mitochondrial dynamics and mitophagy

Acute upper‐body RE was performed immediately after maximal unilateral knee eccentric muscle contractions exercise to determine the role of an acute RE‐induced hormonal changes on markers of mitochondrial dynamics and mitophagy. We performed bivariate correlation analysis investigating correlations between testosterone, growth hormone, and cortisol AUC and the mean protein or gene expression changes from 12 and 24 h for mitochondrial dynamics and mitophagy markers.

**FIGURE 4:**
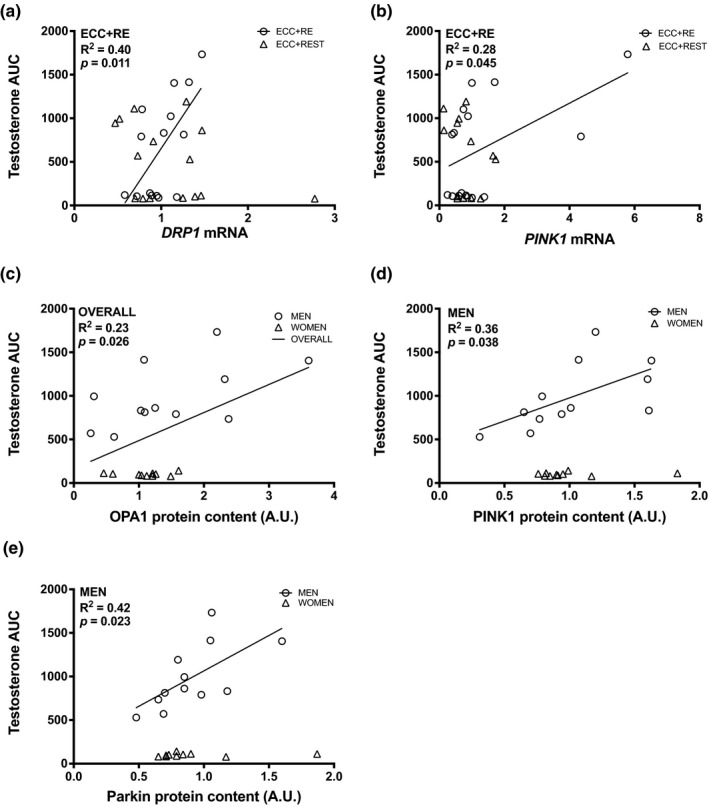
Bivariate correlational analysis for gene expression of (a) *DRP1*, (b) *PINK1* and the protein expression of (c) OPA1, (d) PINK1, and (e) Parkin with testosterone area under the curve. The average of 12 and 24 h were used for gene and protein expression in correlational analyses

For ECC + RE, significant positive correlations were observed for testosterone AUC with *DRP1* gene expression (ECC + RE: R^2^ = 0.40, *p* = 0.011; Figure 4a) and with *PINK1* gene expression (ECC + RE: R^2^ = 0.28, *p* = 0.045; Figure 4b). Additionally, for men, significant positive correlations were observed for testosterone AUC with PINK1 protein expression (R^2^ = 0.36, *p* = 0.038; Figure 4d) and Parkin protein expression (R^2^ = 0.42, *p* = 0.023; Figure 4e). Lastly, regardless of sex and condition, a positive correlation was observed for testosterone AUC with OPA1 protein (R^2^ = 0.23, *p* = 0.026; Figure 4c).

**FIGURE 5:**
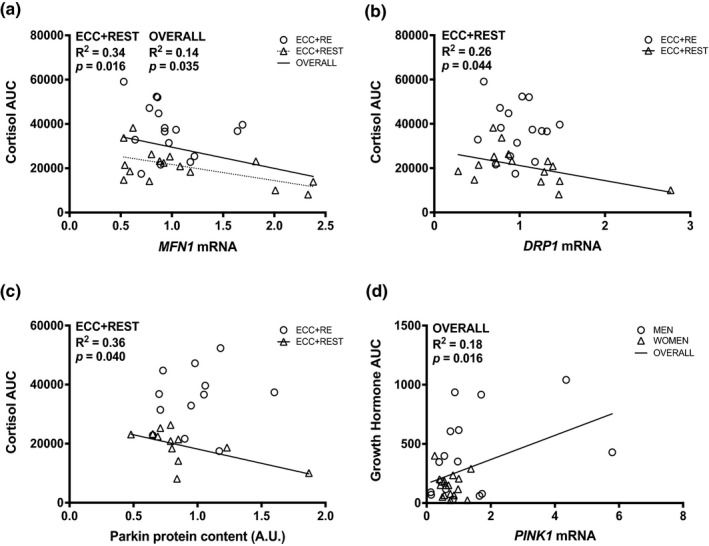
Bivariate correlational analysis for gene expression of (a) *MFN1*, (b) *DRP1*, and protein expression of (c) Parkin with the cortisol area under the curve (AUC) and gene expression of (d) *PINK1* with growth hormone AUC. The average of 12 and 24 h were used for gene and protein expression in correlational analyses

For ECC + REST, a negative correlation was observed for cortisol AUC and *MFN1* gene expression (R^2^ = 0.34, *p* = 0.016; Figure 5a), *DRP1* gene expression (R^2^ = 0.26, *p* = 0.044; Figure 5b.) and Parkin protein expression (R^2^ = 0.36, *p* = 0.040; Figure 5c). Regardless of sex and condition, a positive correlation was observed for growth hormone AUC and *PINK1* gene expression (R^2^ = 0.18, *p* = 0.016; Figure 5d).

## 3 DISCUSSION

The purpose of this study was to examine the effect of acute RE‐induced hormonal response on mitochondrial dynamics (OPA1, MFN1, and DRP1) and mitophagy (PINK1 and Parkin) markers after lower limb muscle damage. The major and novel findings are that the transient increase in RE‐induced hormones appeared only to impact mitochondrial dynamics and mitophagy‐related gene expression in men, where the transcript abundance for *DRP1*, *MFN1*, and *PINK1* was increased. In addition, Parkin protein, a marker of mitophagy, decreased in men after muscle damage alone, whereas, in women, mitophagy was promoted after muscle damage regardless of the hormonal changes. Furthermore, increased OPA1 protein, a marker of IMM fusion, was promoted during the first 24 h after muscle damage regardless of the systemic hormonal changes in both sexes. Combined, these findings indicated that a sex‐specific mitophagy response and mitochondrial IMM fusion were likely promoted after muscle damage. Importantly, the acute RE‐induced increase in hormones could promote the transcriptional capacity for genes controlling fission, OMM fusion, and mitophagy for men, but does not appear to promote these processes for women.

In response to eccentric exercise‐induced muscle damage, regardless of sex and condition, IMM fusion protein OPA1 increased from baseline to 12 and 24 h; whereas, *OPA1* gene expression was decreased at 12 h in men and was lower than women. To date, the effect of eccentric exercise‐induced muscle damage on mitochondrial fusion and fission is not known. With a bout of whole‐body RE, including leg press and leg extension exercises (3 sets of 10–12 repetitions with the rate of perceived exertion (RPE) between 7 and 9 out of 10), Mesquita et al. reported an increase in OPA1 protein at 24 h with no change in DRP1 and MFN protein in skeletal muscle of untrained older men and women (Mesquita et al., 2020). These results were aligned with the current study. Although the mechanical stress differs between traditional RE and maximal eccentric exercise, it is plausible that unaccustomed heavy RE could also result in mitochondrial damage. As demonstrated by Liu and Li (Liu & Li, 2014), exercise‐induced muscle damage can result in cristae degeneration, and together with an increase in OPA1 and no change in MFN1 from the current study, these results could suggest that mitochondria underwent IMM repair attempting to prevent apoptosis to maintain mitochondrial function (Liu & Li, 2014). As a result, OMM fusion might not have been required for remodeling. This explanation has been illustrated by Frezza et al. (2006) who demonstrated that OPA1 is critical to mitochondrial IMM remodeling in response to apoptotic signaling without MFN1 in an in vitro model (Frezza et al., 2006). These results could indicate the initial role of OPA1 in maintaining mitochondrial function after exercise‐induced muscle damage.

Damaged mitochondria are removed by PINK1/Parkin‐mediated mitophagy (Durcan & Fon, 2015). In the present study, Parkin protein expression was decreased at 24 h in men with muscle damage alone; whereas, expression was promoted for women at 24 h after muscle damage regardless of hormonal changes. Furthermore, in the present study, the absence of a change in PINK1 protein suggested an equilibrium between PINK1 degradation and accumulation which occurs when the mitochondrial membrane potential is maintained and disrupted, respectively (Jin et al., 2010). In the context of muscle damage, reactive oxygen species (ROS) are generated (He et al., 2016; Steinbacher & Eckl, 2015) leading to oxidative damage and mitochondrial dysfunction if ROS production exceeds mitochondrial antioxidant capacity (Bhatti et al., 2017). Marzani et al. demonstrated a sex‐specific difference in defending against oxidative stress, where women have a greater ability to defend against oxidative stress than men in skeletal muscle (Marzani et al., 2004). Potentially, more efficient removal of damaged mitochondria for women (indicated by the increase in Parkin in the current study) could reduce ROS (Bhatti et al., 2017; Bin‐Umer et al., 2014) accumulated in the skeletal muscle.

The reduction in Parkin protein after muscle damage has not been well established but has been shown in exercise with aging or high‐fat diet models. Parkin protein decreased in skeletal muscle of aged compared to young mice regardless of exercise (Chen et al., 2018) and in the heart of mice fed a high‐fat diet compared to a low‐fat diet (Thomas et al., 2019). Thomas et al. further conducted polysome profiling to examine potential mechanisms underlying decreased Parkin in the heart, and their results suggested that there was less *Parkin* mRNA in the translating fraction than the non‐translating fraction in mice with a high‐fat diet (Thomas et al., 2019). However, it is important to confirm these findings in skeletal muscle as a decrease in Parkin protein could interfere with mitophagy and maintain functional mitochondria. In addition, the decrease in Parkin found only for ECC + REST but not for ECC + RE in men could indicate that the upper‐body RE initiated the release of hormones or growth factors that could plausibly regulate Parkin translation.

Similar to other cellular processes, circulating growth factors, such as hormones, are essential in regulating nuclear genes that encode mitochondrial transcription factors in maintaining mitochondrial biogenesis (Psarra et al., 2006; Usui et al., 2014). Of particular interest, Pronsato et al. demonstrated that incubating C2C12 cells with a physiological dose of testosterone increased gene expression of mitochondrial transcription factors such as NRF‐1 and OPA1 (downstream of NRF‐1) (Pronsato et al., 2020). To illustrate the role of RE‐induced hormones on mitochondria in response to muscle damage, we incorporated upper‐body RE aimed to induce an acute hormonal response that differs between sexes, and previously published results showed that the testosterone AUC was greater with the ECC + RE than ECC + REST (Luk et al., 2021). Our results were in accordance with the findings of Pronsato et al. (2020) as we found that the inclusion of upper‐body RE, inducing an acute testosterone response, immediately after muscle damage increased transcript abundance of *MFN1*, *DRP1*, and *PINK1* from baseline to 12 h for men but not for women. In the context of muscle damage, the associated mitochondrial cristae degeneration (Liu & Li, 2014) could have decreased antioxidant capacity that could have played a role in changes noticed only in men. Thus, reasons for increases not observed in women could possibly be due to mitochondria in human skeletal muscle having a greater capacity to defend against oxidative stress in women than in men (Marzani et al., 2004). Although speculative, the increase in abundance for *MFN1*, *DRP1*, and *PINK1* in the ECC + RE group for men only could potentially be beneficial if the mitochondria cristae are beyond repair.

Additionally, bivariate correlation analysis was used to illustrate the relationship of RE‐induced or basal testosterone, growth hormone, and cortisol with factors that are critical to mitochondrial quality. In line with findings from Pronsato et al. (2020) on the positive effect of testosterone on genes regulating mitochondrial quality, our results indicated that, regardless of condition, testosterone AUC was positively correlated with PINK1 and Parkin protein expression for men only. Furthermore, regardless of sex, testosterone AUC was positively correlated with *PINK1* and *DRP1* gene expression for the ECC + RE condition. Lastly, in accordance with our observed increase in OPA1 protein at 12 and 24 h after muscle damage, our results indicated that testosterone AUC was positively correlated with overall OPA1 protein expression. Similarly, in response to endurance training after a period of testosterone supplementation, gene expression for *PINK1*, *OPA1*, *DRP1*, and protein expression for DRP1 and MFN2 were greater than exercise without testosterone supplementation in aged male mice (Guo et al., 2012). Combined, these results indicate that, for men, testosterone could play an essential role in maintaining mitochondrial quality in response to exercise stress.

Growth hormone AUC was positively associated with PINK1 protein expression and cortisol AUC was negatively associated with *MFN1* gene expression regardless of sex and condition. Furthermore, cortisol AUC for ECC + REST was also negatively associated with the gene expression of *MFN1*, *DRP1*, and the protein expression Parkin. The understanding of growth hormone and cortisol on mitochondrial dynamics and mitophagy after exercise in skeletal muscle is limited. In aged rats without growth hormone supplementation, NRF‐1 was lower than young rats and aged rats with growth hormone supplementation (Brioche et al., 2014), in which increased NRF‐1 upregulated mitophagy markers (i.e., PINK1 and Parkin) (Lu et al., 2020). In liver cells, dexamethasone incubation resulted in increased DRP1 protein and decreased MFN1/2 protein (Hernández‐Alvarez et al., 2013). However, further studies are required to further our understanding of cortisol and growth hormone’s role in mitochondrial dynamics and mitophagy in skeletal muscle. Overall, our results from the correlative analysis suggest that basal and RE‐induced transient increases in circulating testosterone, growth hormone, and cortisol concentration could influence the mitochondrial response following muscle damage.

In conclusion, the findings of this study demonstrated that muscle damage promotes markers of IMM fusion (OPA1) in both sexes; whereas, markers of mitophagy (Parkin) were promoted in women but reduced in men after muscle damage. Interestingly, when adding upper‐body RE to elicit an acute hormonal response after muscle damage, the transcript abundance of *DRP1*, *MFN1*, and *PINK1* increased in the damaged muscle tissue for men only. Thus, muscle damage causes both muscle and mitochondrial damage that appears to affect the mitophagic process differently in men and women. Based on these findings, the circulating hormonal increases (e.g., testosterone), despite being transient, appeared to be important for mitochondrial repair after muscle damage in men.

## 4 MATERIALS AND METHODS

### 4.1 Ethical approval

This study was approved by the University Institutional Review Board and adhered to the Declaration of Helsinki. All participants provided written informed consent to be part of the study.

### 4.2 Overall study design

This study employed a cross‐over, randomized study design as depicted in Figure 1. The details of the study design, protocol, and participants’ descriptive data have been published (Luk et al., 2021). Briefly, vastus lateralis samples from 16 young untrained men (*n* = 8) and women (*n* = 8) were utilized in this report. Muscle samples were collected at BL, 12 and 24 h after performing eccentric muscle damaging exercises followed by acute upper‐body RE (completed in about 20 min) or seated rest for 20 min. Muscle samples were analyzed for intramuscular gene expression of *OPA1*, *MFN1*, *DRP1*, *PINK1*, and *Parkin* and protein expression of OPA1, DRP1, PINK1, and Parkin.

### 4.3 Participants

For this investigation, we analyzed muscle samples from a subset of participants (men: *n* = 8; women: *n* = 8), and the details of the study design (Luk et al., 2019) and participants’ descriptive data (Luk et al., 2021) have been previously reported. Participants included eight untrained young men (age: 22 ± 3 years; lean mass: 58.4 ± 6.8 kg; body fat: 23.2 ± 9.2%) and women (age: 20 ± 1 years; lean mass: 36.5 ± 5.1 kg; body fat %: 36.3 ± 8.2%) (Luk et al., 2021). Participants provided medical and activity history forms to determine eligibility for participation. Eligible participants provided written informed consent, and to be included, participants must have been free from any musculoskeletal injuries, did not participate in any form of resistance or endurance training in the past year, and did not consume any hormonal substances or medications (i.e., anabolic steroids, growth hormone, or glucocorticoids). Women were eumenorrheic and without oral contraceptives for at least three months.

### 4.4 Anthropometric measurements, familiarization, and 1‐repetition maximum test

Briefly, anthropometric measurements, familiarization of all exercises, and one‐repetition maximum (1‐RM) testing were performed approximately one week before the exercise protocol. First, height, weight, and body composition (dual‐energy X‐ray absorptiometry) were measured, then participants were familiarized with the unilateral eccentric knee extension protocol for both legs. Participants were instructed to use only light resistance during familiarization to avoid muscle damage at that time. Next, participants were instructed in and demonstrated the proper technique for the exercises included in the upper‐body RE protocol (i.e., free weight bench press, bench row, and seated overhead press exercises). Then, 1‐RM was measured for each upper‐body exercise.

#### 4.4.1 Exercise visits

The details of the exercise visits have been previously described (Luk et al., 2019). Briefly, participants completed two identical visits of the maximal unilateral eccentric knee extension exercise (ECC) aimed to induce muscle damage, with the contralateral leg exercise on the second visit, and results showed an increase in myoglobin concentration (Luk et al., 2021). Then, participants either completed upper‐body RE (ECC + RE) or were seated for 20‐min (ECC + REST). With the upper‐body RE, there was a greater increase in circulating testosterone, growth hormone, and cortisol than 20‐min seated rest (Luk et al., 2021, 2019). Muscle samples were collected from the vastus lateralis of the exercising leg at BL, 12 and 24 h after the exercise visit for measurement of intramuscular gene expression (*OPA1*, *MFN1*, *DRP1*, *PINK1*, and *Parkin*) and protein expression (OPA1, DRP1, PINK1, and Parkin). The exercise visits were conducted for women during the early follicular phase of two consecutive menstrual cycles to control for estrogen variation. The two exercise visits were separated by approximately 28 days for men and women to maintain consistency of timing between visits.

Participants completed at least a 10‐h fast (except for water) prior to each exercise visit. Hydration status was assessed using a refractometer at the start of all visits. If urine specific gravity was ≥1.020, they were required to consume 16 oz of water and rest for 10 min before blood collection. Following the 10‐min rest, a cannula was inserted in a superficial vein in the forearm, and a BL blood sample was collected. The BL muscle sample was collected immediately thereafter via micro biopsy.

After completing the dynamic warm‐up, participants completed maximal unilateral eccentric knee extensions (8 sets of 10 repetitions; 3 min rest between sets). A blood sample was collected immediately (IMD) after completion. Participants then either completed ECC + RE or ECC + REST. For ECC + RE, after IMD blood collection, participants immediately performed seated bench press, bench row, and overhead press (4 sets of 10 repetitions at 80% of 1‐RM; 1‐min between sets and exercises) (Luk et al., 2019). Our results indicated that this exercise protocol elicited a RE‐induced hormonal response in untrained participants (Luk et al., 2021). If participants could not complete repetitions due to muscle fatigue, minimal assistance was provided during the concentric portion of the exercise and for the remaining repetitions in the set. The load was then reduced so that the remaining sets could be completed without assistance. Blood samples were collected IMD and 15, 30, and 60 min after ECC + RE or ECC + REST.

Muscle samples were collected 12 and 24 h after completing the exercise protocol (±30 min). A 3 and 10 h fast were required prior to the 12 and 24 h muscle collection, respectively. To minimize the potential confounding variables from diet intake, participants recorded the mealtime, type, and amount of food and drink following the exercise visit and were instructed to follow the exact diet for the second exercise visit. Consumption of alcohol and nonsteroidal anti‐inflammatory drugs was prohibited 48 h before and 24 h after the exercise visit.

### 4.5 Muscle biopsies

The details of the muscle biopsy have been previously described (Luk et al., 2019). Muscle biopsies were obtained from the vastus lateralis at BL, 12 and 24 h from the leg that performed the eccentric muscle damaging knee extension exercise using micro biospy. First, betadine was used to clean the leg, followed by a local anesthetic (1% lidocaine w/o epinephrine) injection into the vastus lateralis. Next, a Pro‐Mag 14‐gauge muscle biopsy needle (Angiotech, Gainesville, FL) was used to obtain muscle samples from three separate sites (3 cm apart) for the three‐time points. Muscle samples were flash‐frozen in liquid nitrogen once obtained. Sterile gauze was used to cover and compress the incision site to prevent bleeding before applying an adhesive bandage over the site. Participants were provided with written instructions and supplies for biopsy site care.

### 4.6 Serum hormone analyses

These data were a subset of those published previously (Luk et al., 2019). Serum samples from BL, IMD, 15, 30 and 60 were analyzed for testosterone (EIA1559; intra‐assay % CV = 7.12–9.85; inter‐assay % CV = 9.00), growth hormone (EIA1787; intra‐assay % CV = 8.02–14.23; inter‐assay % CV = 10.32), and cortisol (EIA1887R; intra‐assay % CV = 3.12–9.45; inter‐assay % CV = 5.95) concentrations using commercially available ELISAs (DRG Internation al Inc. Springfield, NJ).

### 4.7 Muscle tissue homogenization and RNA isolation

The details of the muscle tissue homogenization and RNA isolation have been previously described (Luk et al., 2019). Briefly, muscle samples were isolated using RNeasy Fibrous Tissue Mini Kit Cat. # 74704, Qiagen, Germantown, MD) using the protocol stated by the manufacturer. First, the homogenizing buffer was added to the muscle sample, which was then homogenized using a bead‐based tissue homogenizer. Samples were then incubated at 55°C for 10 min and centrifuged. Next, the supernatant was isolated, mixed with ethanol, and added to the RNeasy Mini Column and recentrifuged. DNase stock solution was then added to the RNeasy membrane and incubated, followed by adding Buffer RW1 to the RNeasy membrane, which was centrifuged, and the flow‐through was discarded. After drying the membrane, the RNeasy column was placed in a new 1.5 mL tube, and RNase‐free water was added and RNA eluted.

### 4.8 Muscle gene expression

mRNA expression analyses were performed using our previously published methods (Luk et al., 2021). Briefly, muscle samples were analyzed for intramuscular gene expression of *DRP1*, *OPA1*, *MFN1*, *PINK1*, and *Parkin* at BL, 12 and 24 h post‐exercise using real‐time PCR, which was performed using # 2 ABI PRISM70500 Sequence Detection System (Applied Biosystems) with iTag SYBR Green Supermix (Bio‐rad) and was normalized to Human beta 2‐microglobulin (*B2 M*). Relative fold change in transcript abundance was determined using the 2^−ΔΔCt^ method.

### 4.9 Western immunoblot analyses

The details of the western blot procedure have been previously described (Luk et al., 2019). Briefly, muscle samples were homogenized, agitated, and centrifuged. The supernatant was isolated and analyzed for protein content using microplate (Take 3, Bio‐Tek, Winoosko, VT) spectrophotometry (Epoch, Bio‐Tek) and was stored at −80°C until western blot analysis. Supernatant and loading buffer were sonicated and heated, and 45 *µ*g of protein was loaded into 20% polyacrylamide gel (BioRad, Hercules, CA) in duplicate and separated at 120 V for 45 min at room temperature. For each participant, samples from each time point and condition were loaded on the same gel and gels were run in duplicate. Each membrane was stripped no more than twice and the density of each protein band was normalized to the density of GAPDH (housekeeping protein) from the same lane (Pillai‐Kastoori et al., 2020). Samples were then transferred electrophoretically to a PVDF membrane at 70 V at 4°C for 2.5 h. Next, 5% non‐fat milk powder was used to block the membranes for 1 h, and after that, the membrane was incubated with primary antibodies against OPA1 (1:1000; Cell Signaling, 80471S; 80 kDa), DRP1 (1:1000; Novus, NB110‐55288; 80 kDa), PINK1 (1:2000; Novus, NBP1‐49678; 55 kDa), Parkin (1:1000; Cell Signaling, 2132S; 55 kDa), and GAPDH (1:5000; Santa Cruz, sc‐365062; 37 Membranes were stripped) with 3% non‐fat milk at 4°C overnight. After overnight incubation, the membranes blocked with mouse monoclonal primary antibodies were blocked with secondary anti‐mouse IgG (1:1000; Santa Cruz, sc‐2005), and membranes blocked with rabbit monoclonal primary antibody were blocked with anti‐rabbit IgG (1:5000; Santa Cruz, sc‐2004) along with Anti‐HRP (1:20,000; BioRad, 161–0380) for 1 h at room temperature with 5% non‐fat milk. Chemiluminescent substrate (WesternBright Sirius HRP substrate Advanta, Menlo Park, CA) and the C‐Digit imaging system (Li‐Cor, Lincoln, NE) were used to visualize the stained protein bands. Image Studio Digits Ver 4.0 (Li‐Cor) was used for band densitometry. Membranes were stripped using 5X Western Reprobe for 60 min at room temperature with agitation. To analyze the protein content, each protein band was normalized to its respective GAPDH band. Then, the protein content at BL, 12 and 24 h was further normalized to men ECC + REST at BL.

### 4.10 Statistical analyses

SPSS (IBM version 26; Armonk, NY: IBM Corp) was used for all statistical analyses. Log10 transformation was used where the assumption of normality was violated. Gene and protein expression were analyzed using three‐way ANOVA (time × sex × condition) with repeated measures on condition and time. Bonferroni post hoc tests were used for pairwise comparisons. In addition, bivariate Pearson correlations were used to test associations between gene and protein expression of mitochondrial dynamics and mitophagy markers to testosterone, growth hormone, and cortisol AUC. The statistical significance was set at *p* ≤ 0.05.

## CONFLICT OF INTEREST

No conflicts of interest, financial or otherwise, are declared by the authors. Additionally, the results of the study are presented clearly, honestly, and without fabrication, falsification, or inappropriate data manipulation.

## References

[phy215230-bib-0001] Bhatti, J. S. , Bhatti, G. K. , & Reddy, P. H. (2017). Mitochondrial dysfunction and oxidative stress in metabolic disorders — A step towards mitochondria based therapeutic strategies. Biochimica Et Biophysica Acta (BBA) ‐ Molecular Basis of Disease, 1863(5), 1066–1077. 10.1016/j.bbadis.2016.11.010 27836629 PMC5423868

[phy215230-bib-0002] Bin‐Umer, M. A. , McLaughlin, J. E. , Butterly, M. S. , McCormick, S. , & Tumer, N. E. (2014). Elimination of damaged mitochondria through mitophagy reduces mitochondrial oxidative stress and increases tolerance to trichothecenes. Proceedings of the National Academy of Sciences, 111(32), 11798–11803. 10.1073/pnas.1403145111 PMC4136610 25071194

[phy215230-bib-0003] Brioche, T. , Kireev, R. A. , Cuesta, S. , Gratas‐Delamarche, A. , Tresguerres, J. A. , Gomez‐Cabrera, M. C. , & Vina, J. (2014). Growth hormone replacement therapy prevents sarcopenia by a dual mechanism: improvement of protein balance and of antioxidant defenses. Journals of Gerontology Series A, Biological Sciences and Medical Sciences, 69(10), 1186–1198. 10.1093/gerona/glt187 24300031

[phy215230-bib-0004] Chen, C. C. W. , Erlich, A. T. , Crilly, M. J. , & Hood, D. A. (2018). Parkin is required for exercise‐induced mitophagy in muscle: Impact of aging. American Journal of Physiology. Endocrinology and Metabolism, 315(3), E404–E415. 10.1152/ajpendo.00391.2017 29812989

[phy215230-bib-0005] Chen, H. , Vermulst, M. , Wang, Y. E. , Chomyn, A. , Prolla, T. A. , McCaffery, J. M. , & Chan, D. C. (2010). Mitochondrial fusion is required for mtDNA stability in skeletal muscle and tolerance of mtDNA mutations. Cell, 141(2), 280–289. 10.1016/j.cell.2010.02.026 20403324 PMC2876819

[phy215230-bib-0006] Dulac, M. , Leduc‐Gaudet, J. P. , Reynaud, O. , Ayoub, M. B. , Guérin, A. , Finkelchtein, M. , Hussain, S. N. A. , & Gouspillou, G. (2020). Drp1 knockdown induces severe muscle atrophy and remodelling, mitochondrial dysfunction, autophagy impairment and denervation. Journal of Physiology, 598(17), 3691–3710. 10.1113/JP279802 32539155

[phy215230-bib-0007] Durcan, T. M. , & Fon, E. A. (2015). The three ‘P’s of mitophagy: PARKIN, PINK1, and post‐translational modifications. Genes & Development, 29(10), 989–999. 10.1101/gad.262758.115 25995186 PMC4441056

[phy215230-bib-0008] Frezza, C. , Cipolat, S. , Martins de Brito, O. , Micaroni, M. , Beznoussenko, G. V. , Rudka, T. , Bartoli, D. , Polishuck, R. S. , Danial, N. N. , De Strooper, B. , & Scorrano, L. (2006). OPA1 controls apoptotic cristae remodeling independently from mitochondrial fusion. Cell, 126(1), 177–189. 10.1016/j.cell.2006.06.025 16839885

[phy215230-bib-0010] Gouspillou, G. , Godin, R. , Piquereau, J. , Picard, M. , Mofarrahi, M. , Mathew, J. , Purves‐Smith, F. M. , Sgarioto, N. , Hepple, R. T. , Burelle, Y. , & Hussain, S. N. A. (2018). Protective role of Parkin in skeletal muscle contractile and mitochondrial function: Parkin is essential for optimal muscle and mitochondrial functions. Journal of Physiology, 596(13), 2565–2579. 10.1113/JP275604 29682760 PMC6023825

[phy215230-bib-0011] Guo, W. , Wong, S. , Li, M. , Liang, W. , Liesa, M. , Serra, C. , Jasuja, R. , Bartke, A. , Kirkland, J. L. , Shirihai, O. , & Bhasin, S. (2012). Testosterone plus low‐intensity physical training in late life improves functional performance, skeletal muscle mitochondrial biogenesis, and mitochondrial quality control in male mice. PLoS One, 7(12), e51180. 10.1371/journal.pone.0051180 23240002 PMC3519841

[phy215230-bib-0012] He, F. , Li, J. , Liu, Z. , Chuang, C. ‐C. , Yang, W. , & Zuo, L. (2016). Redox mechanism of reactive oxygen species in exercise. Frontiers in Physiology, 7, 10.3389/fphys.2016.00486 PMC5097959 27872595

[phy215230-bib-0013] Hernández‐Alvarez, M. I. Paz, J. C. , Sebastián, D. , Muñoz, J. P. , Liesa, M. , Segalés, J. , Palacín, M. , & Zorzano, A. (2013). Glucocorticoid modulation of mitochondrial function in hepatoma cells requires the mitochondrial fission protein Drp1. Antioxidants & Redox Signaling, 19(4), 366–378. 10.1089/ars.2011.4269 22703557 PMC3700019

[phy215230-bib-0014] Hoitzing, H. , Johnston, I. G. , & Jones, N. S. (2015). What is the function of mitochondrial networks? A theoretical assessment of hypotheses and proposal for future research. BioEssays: News and Reviews in Molecular, Cellular and Developmental Biology, 37(6), 687–700. 10.1002/bies.201400188 25847815 PMC4672710

[phy215230-bib-0015] Hu, B. , Li, H. , & Zhang, X. (2021). A balanced act: The effects of GH–GHR–IGF1 Axis on mitochondrial function. Frontiers in Cell and Developmental Biology, 9, 630248. 10.3389/fcell.2021.630248 33816476 PMC8012549

[phy215230-bib-0016] Jin, S. M. , Lazarou, M. , Wang, C. , Kane, L. A. , Narendra, D. P. , & Youle, R. J. (2010). Mitochondrial membrane potential regulates PINK1 import and proteolytic destabilization by PARL. Journal of Cell Biology, 191(5), 933–942. 10.1083/jcb.201008084 21115803 PMC2995166

[phy215230-bib-0017] Kitaoka, Y. , Ogasawara, R. , Tamura, Y. , Fujita, S. , & Hatta, H. (2015). Effect of electrical stimulation‐induced resistance exercise on mitochondrial fission and fusion proteins in rat skeletal muscle. Applied Physiology, Nutrition and Metabolism, 40(11), 1137–1142. 10.1139/apnm-2015-0184 26513006

[phy215230-bib-0018] Liesa, M. , & Shirihai, O. S. (2013). Mitochondrial dynamics in the regulation of nutrient utilization and energy expenditure. Cell Metabolism, 17(4), 491–506. 10.1016/j.cmet.2013.03.002 23562075 PMC5967396

[phy215230-bib-0019] Liu, X. , & Li, Y. (2014). Cytoskeletal vimentin protein expression in rats with exhaustive eccentric exercise injury. Chinese Journal of Tissue Engineering Research, 18(18), 2880–2885.

[phy215230-bib-0020] Lu, Y. , Ding, W. , Wang, B. , Wang, L. , Kan, H. , Wang, X. , Wang, D. , & Zhu, L. (2020). Positive regulation of human PINK1 and Parkin gene expression by nuclear respiratory factor 1. Mitochondrion, 51, 22–29. 10.1016/j.mito.2019.12.002 31862413

[phy215230-bib-0021] Luk, H. Y. , Levitt, D. E. , Appel, C. , & Vingren, J. L. (2021). Sex dimorphism in muscle damage‐induced inflammation. Medicine & Science in Sports & Exercise, 53(8), 1595–1605. Publish Ahead of Print. doi:10.1249/MSS.0000000000002628 34261990

[phy215230-bib-0022] Luk, H.‐Y. , Levitt, D. E. , Boyett, J. C. , Rojas, S. , Flader, S. M. , McFarlin, B. K. , & Vingren, J. L. (2019). Resistance exercise‐induced hormonal response promotes satellite cell proliferation in untrained men but not in women. American Journal of Physiology‐Endocrinology and Metabolism, 317(2), E421–E432. 10.1152/ajpendo.00473.2018 31237450

[phy215230-bib-0023] Marzani, B. , Pansarasa, O. , & Marzatico, F. (2004). “Oxidative stress” and muscle aging: influence of age, sex, fiber composition and function. Basic and Applied Myology, 14(1), 37–44.

[phy215230-bib-0024] Mesquita, P. H. C. , Lamb, D. A. , Parry, H. A. , Moore, J. H. , Smith, M. A. , Vann, C. G. , Osburn, S. C. , Fox, C. D. , Ruple, B. A. , Huggins, K. W. , Fruge, A. D. , Young, K. C. , Kavazis, A. N. , & Roberts, M. D. (2020). Acute and chronic effects of resistance training on skeletal muscle markers of mitochondrial remodeling in older adults. Physiological Reports, 8, e14526. 10.14814/phy2.14526 32748504 PMC7399374

[phy215230-bib-0025] Narendra, D. , Kane, L. A. , Hauser, D. N. , Fearnley, I. M. , & Youle, R. J. (2010). p62/SQSTM1 is required for Parkin‐induced mitochondrial clustering but not mitophagy; VDAC1 is dispensable for both. Autophagy, 6(8), 1090–1106. 10.4161/auto.6.8.13426 20890124 PMC3359490

[phy215230-bib-0100] Pillai‐Kastoori, L. , Schutz‐Geschwender, A. R. , & Harford, J. A. (2020). A systematic approach to quantitative Western blot analysis. Analytical Biochemistry, 593, 113608. 10.1016/j.ab.2020.113608 32007473

[phy215230-bib-0026] Pronsato, L. , Milanesi, L. , & Vasconsuelo, A. (2020). Testosterone induces up‐regulation of mitochondrial gene expression in murine C2C12 skeletal muscle cells accompanied by an increase of nuclear respiratory factor‐1 and its downstream effectors. Molecular and Cellular Endocrinology, 500, 110631. 10.1016/j.mce.2019.110631 31676390

[phy215230-bib-0027] Psarra, A. M. G. , Solakidi, S. , & Sekeris, C. E. (2006). The mitochondrion as a primary site of action of regulatory agents involved in neuroimmunomodulation. Annals of the New York Academy of Sciences, 1088, 12–22. 10.1196/annals.1366.019 17192553

[phy215230-bib-0028] Sebastián, D. , Palacín, M. , & Zorzano, A. (2017). Mitochondrial dynamics: coupling mitochondrial fitness with healthy aging. Trends in Molecular Medicine, 23(3), 201–215. 10.1016/j.molmed.2017.01.003 28188102

[phy215230-bib-0029] Sin, J. , Andres, A. M. , Taylor, D. J. R. , Weston, T. , Hiraumi, Y. , Stotland, A. , Kim, B. J. , Huang, C. , Doran, K. S. , & Gottlieb, R. A. (2016). Mitophagy is required for mitochondrial biogenesis and myogenic differentiation of C2C12 myoblasts. Autophagy, 12(2), 369–380. 10.1080/15548627.2015.1115172 26566717 PMC4836019

[phy215230-bib-0030] Steinbacher, P. , & Eckl, P. (2015). Impact of oxidative stress on exercising skeletal muscle. Biomolecules, 5(2), 356–377. 10.3390/biom5020356 25866921 PMC4496677

[phy215230-bib-0031] Tezze, C. , Romanello, V. , Desbats, M. A. , Fadini, G. P. , Albiero, M. , Favaro, G. , Ciciliot, S. , Soriano, M. E. , Morbidoni, V. , Cerqua, C. , Loefler, S. , Kern, H. , Franceschi, C. , Salvioli, S. , Conte, M. , Blaauw, B. , Zampieri, S. , Salviati, L. , Scorrano, L. , & Sandri, M. (2017). Age‐associated loss of OPA1 in muscle impacts muscle mass, metabolic homeostasis, systemic inflammation, and epithelial senescence. Cell Metabolism, 25(6), 1374–1389.e6. 10.1016/j.cmet.2017.04.021 28552492 PMC5462533

[phy215230-bib-0032] Thomas, A. , Marek‐Iannucci, S. , Tucker, K. C. , Andres, A. M. , & Gottlieb, R. A. (2019). Decrease of cardiac parkin protein in obese mice. Front Cardiovasc Med, 6, 191. 10.3389/fcvm.2019.00191 32039238 PMC6984192

[phy215230-bib-0033] Usui, T. , Kajita, K. , Kajita, T. , Mori, I. , Hanamoto, T. , Ikeda, T. , Okada, H. , Taguchi, K. , Kitada, Y. , Morita, H. , Sasaki, T. , Kitamura, T. , Sato, T. , Kojima, I. , & Ishizuka, T. (2014). Elevated mitochondrial biogenesis in skeletal muscle is associated with testosterone‐induced body weight loss in male mice. FEBS Letters, 588(10), 1935–1941. 10.1016/j.febslet.2014.03.051 24726723

[phy215230-bib-0034] Vingren, J. L. , Kraemer, W. J. , Ratamess, N. A. , Anderson, J. M. , Volek, J. S. , & Maresh, C. M. (2010). Testosterone physiology in resistance exercise and training: the up‐stream regulatory elements. Sports Medicine (Auckland, N. Z.), 40(12), 1037–1053. 10.2165/11536910-000000000-00000 21058750

[phy215230-bib-0035] Weiss, L. , Cureton, K. , & Thompson, F. (1983). Comparison of serum testosterone and androstenedione responses to weight lifting in men and women. European Journal of Applied Physiology, 50, 413–419. 10.1007/BF00423247 6683165

[phy215230-bib-0036] Ying, J. , Cen, X. , & Yu, P. (2021). Effects of eccentric exercise on skeletal muscle injury: From an ultrastructure aspect: A review. Phys Act Health, 5(1), 15–20. 10.5334/paah.67

[phy215230-bib-0037] Youle, R. J. , & van der Bliek, A. M. (2012). Mitochondrial fission, fusion, and stress. Science, 337(6098), 1062–1065. 10.1126/science.1219855 22936770 PMC4762028

[phy215230-bib-0038] Yu, S. B. , & Pekkurnaz, G. (2018). Mechanisms orchestrating mitochondrial dynamics for energy homeostasis. Journal of Molecular Biology, 430(21), 3922–3941. 10.1016/j.jmb.2018.07.027 30089235 PMC6186503

